# A decade of migrasome research: biogenesis, physiological functions, and disease implications

**DOI:** 10.1038/s41422-025-01153-0

**Published:** 2025-08-22

**Authors:** Jinqiang Yu, Li Yu

**Affiliations:** 1 State Key Laboratory of Membrane Biology, Beijing, China; 2 Beijing Frontier Research Center for Biological Structure, Beijing, China; 3 School of Life Sciences, Tsinghua University, Beijing, China; 4 Tsinghua University–Peking University Joint Center for Life Sciences, Beijing, China

**Keywords:** Cell biology, Organelles

## Abstract

Since their first report a decade ago, our understanding of migrasomes — specialized organelles initially identified in migrating cells—has advanced considerably. Researchers have elucidated key aspects of migrasome biology, including the mechanisms of their biogenesis, their roles in cellular physiology, and their implications in various diseases. Concurrently, the development of a robust toolkit for migrasome analysis has transformed these structures from mere microscopy curiosities into central players in an emerging field with significant impact on cell biology, developmental biology, immunology, and disease pathology. This review provides a comprehensive summary of current insights into migrasome biology, with a particular focus on the molecular mechanisms governing their formation and their established cellular and physiological functions. In addition, we highlight the current challenges and unresolved questions that continue to shape and propel future research in this exciting area of study.

## Introduction

### Discovery of migrasomes

Migrasomes were first reported in this journal in 2015.^1^ While studying normal rat kidney (NRK) cells, Ma et al. observed large membrane-bound structures containing varying numbers of smaller vesicles, some of which appeared densely packed. Due to their resemblance to opened pomegranate, these structures were initially referred to as “pomegranate-like structures.” Scanning electron microscopy further revealed that these structures were connected to cells by long membrane tethers known as retraction fibers. Subsequent mass spectrometry analysis identified tetraspanin-4 (Tspan4) as a highly enriched protein in these structures. This enabled the authors to label them using Tspan4-GFP, uncovering that their formation was dependent on cell migration. As a result, the term “pomegranate-like structures” was replaced with “migrasomes” to reflect their migration-associated origin.

### Classification of migrasomes

Migrasomes were initially classified as organelles due to their intricate structure and topological similarity to cilia, the first recognized organelle.^1,2^ However, subsequent research, particularly observations of neutrophils in vivo, revealed that migrasomes frequently detach from cells and are released into the extracellular environment, such as the bloodstream.^3^ These findings suggest that detached migrasomes share key characteristics with extracellular vesicles (EVs), prompting a reevaluation of whether migrasomes should be redefined as EVs rather than organelles. Adding to this complexity, recent studies have shown that migrasomes also function as localized platforms for exocytosis, enabling the exocytosis of secretory proteins from migrasome while still tethered to the cell via retraction fibers.^4,5^ This dual behavior highlights two distinct roles: migrasomes facilitate localized secretion when attached to the cell, and once detached, they exhibit properties akin to EVs. Given these observations, categorizing migrasomes solely as EVs or as localized exocytosis sites may be overly simplistic. Their unique ability to act as both secretory hubs and extracellular carriers suggests that defining migrasomes as specialized organelles involved in intercellular communication may be a more accurate description.

Regardless of how migrasomes are ultimately classified, once they detach from cells, they fall under the category of large EVs. A frequently raised question is how migrasomes differ from other EV types. It is relatively easy to distinguish migrasomes from exosomes and other small EVs, as migrasomes are much larger and originate from different membrane compartments than small EVs such as exosomes.^1,6^ Although systematic comparisons have primarily been conducted between migrasomes and exosomes — resulting in establishment of a set of protein markers to distinguish exosomes and migrasomes biochemically^7 ^— recent studies on migrasome biogenesis and cargo-loading mechanisms have highlighted several distinguishing features that can be useful for differentiating detached migrasomes from other types of EVs, especially other types of large EVs.^8,9^

Morphologically, detached migrasomes are easily distinguished from large EVs. Unlike other large EVs, such as large oncosomes, apoptotic bodies, or microvesicles, migrasomes exhibit two unique structural characteristics. First, many (though not all) migrasomes retain a short segment of retraction fiber, giving them a distinct tadpole-like appearance.^1,3,5,7^ Second, migrasomes contain intraluminal vesicles (ILVs), a hallmark feature that sets them apart.^1,4^ Biochemically distinguishing large EVs remains challenging, mainly due to limited understanding of their cellular origins, biogenesis, and cargo-loading mechanisms. This lack of knowledge has prevented the establishment of universally accepted marker proteins for different large EV types. For migrasomes, recent studies have uncovered unique biogenesis and cargo-loading mechanisms that distinguish them from other EVs. These findings reveal a distinct set of proteins enriched in migrasomes, which may serve as potential markers for their identification.^3,5,10,11^ Moreover, recent studies have uncovered unique biogenesis and cargo-loading mechanisms in migrasomes that are absent in other EVs. One notable example is the presence of secretory carriers within migrasomes, which actively transport and enrich secretory proteins into migrasomes.^4,12^ There is no report of such mechanisms in other large EVs. Consequently, specific proteins associated with secretory carriers in migrasomes, such as VAMPs, Rabs, and certain secretory proteins (e.g., cytokines and chemokines), may serve as distinguishing markers. Additionally, recent work has shown that neutrophil-derived migrasomes (neu-migrasomes) lack phosphatidylserine (PS) exposure on their surface.^3^ This feature can distinguish migrasomes from neutrophil-derived particles and apoptotic bodies. If the absence of PS exposure is consistent across all migrasomes, it could serve as a distinguishing feature to differentiate migrasomes from certain types of other large EVs (Table 1).

**Table 1:** Characteristics of migrasomes and other EVs

Property	Migrasomes	Exosomes	Microvesicles	Large oncosomes	Apoptotic body
Size (nm)	**~500–3000**	**~30–150**	**100–1000**	**1000–10,000**	**1000–5000**
Cargoes	**Secretory vesicles**; **damaged mitochondria**; mRNA, protein, lipid, etc.	RNA, protein, lipid, polysaccharides, glycan, etc.	ribosomal, centrosomal, and mitochondrial proteins, RNA, lipid, etc.	mRNA, miRNA, protein, lipid, etc.	**DNA fragments**, entire organelles, RNA, etc.
Biogenesis	Plasma membrane, **at the junctions or tips of RFs**	**Multivesicular body (MVB)**	Plasma membrane	Plasma membrane	Plasma membrane, cellular fragments
Lipid component	**Sphingomyelin (SM),** PI(4,5)P2	Cholesterol, **ceramide,** **sphingolipids**, etc.	Cholesterol, diacylglycerol, **exposure of phosphatidylserine**, etc.	**Exposure of phosphatidylserine**, etc.	**Exposure of phosphatidylserine**, etc.
Distinct proteins	**SM synthase2 (SMS2)**; **Tetraspanins (Tspan4),** **Integrins**, PIP5K, Rab35, Synaptotagmin-1 (Syt1), **NDST1,** **PIGK,** **CPQ,** **EOGT**, etc.	**ESCRTs,** **Tetraspanins (CD9, CD63, CD81)**, HSP70, integrins, syntenin 1, flotillins, Rab7, exocyst, etc.	**Matrix metalloproteinases (MMPs)**, integrins, cytoskeleton proteins (actin), selectins, **CD40 ligand,** **lineage markers**, etc.	**Metalloproteinases,** **ARF6**, caveolin-1, GAPDH, LDHB, HSPA5, MDH, GPI, **lineage markers**, etc.	**Annexin V**, MFG-E8, Gas6, histones, etc.
References	^1,4,7,8,24–28,31,39,40^	^84–93^	^84–87,94–100^	^84–87,101,102^	^84–86,103–112^

It is important to note that migrating cells can release multiple types of EVs, leaving a trail of EVs behind them.^13–21^ In 2008, Kriebel et al.^15^ made the first observation of this phenomenon, demonstrating that during chemotaxis in *Dictyostelium* cells, adenylyl cyclase (ACA) accumulates in multivesicular bodies (MVBs) at the rear of the cell. These MVBs then release ACA-containing small EVs, which play a crucial role in organizing cells into head-to-tail arrays during collective migration. A similar mechanism has since been observed in migrating cancer cells and neutrophils, where CD63-positive small EVs are released and serve as guidance signals for neighboring cells.^17,19^ More recently, studies have shown that cells treated with lysosome-damaging agents undergo polarized exocytosis of autolysosomes at their basal membrane. These autolysosomes contain ILVs derived from MVBs and damaged lysosomes, leaving trails of exosomes and lysosomal debris behind migrating cells.^22^ Beyond exosomes and vesicular structures from MVBs or autolysosomes, migrating cells can also release “retractosomes.” These are small, sealed vesicles (with diameters of 50–250 nm) formed by the breakage of retraction fibers during cell migration.^23^

## Cellular, ex vivo, and in vivo models for migrasome studies

**Fig. 1:**
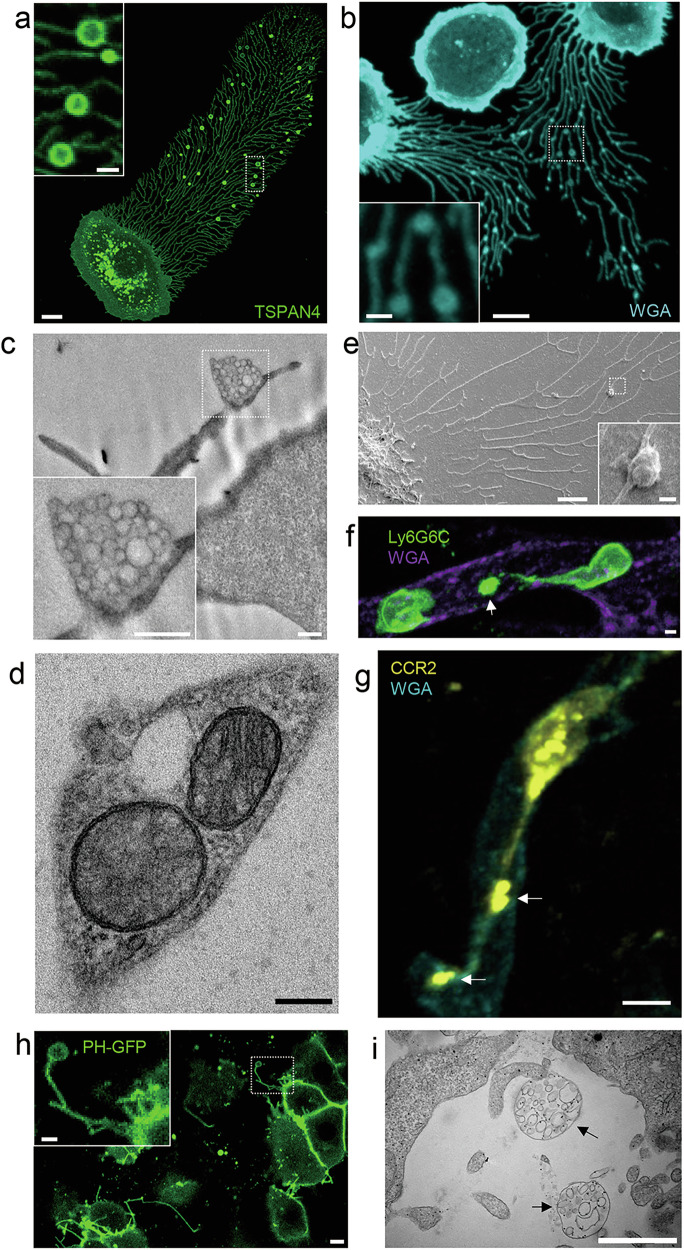
A collection of migrasomes. **a** TSPAN4-GFP overexpressing MCA-205 cancer cell migrated on FN-coated surface. Scale bar, 5 μm. The enlarged box shows migrasomes and retraction fibers. Scale bar, 2 μm. **b** Neutrophil cells isolated from mouse bone marrow were activated with 1 μM fMLP and migrated on a confocal dish. Cells were stained with WGA-647 before imaging. Scale bar, 5 μm. The enlarged box shows migrasomes and retraction fibers. Scale bar, 1 μm. **c** Transmission electron microscopy image of the lowest ultra-thin section (in contact with the culture dish) of a L929 cell. Scale bar, 500 nm. Enlarged migrasome is shown in the down left panels. Scale bar, 500 nm. **d** Representative transmission electron microscopy images of L929 cell treated with 2 mM CCCP. Scale bar, 20 nm. **e** Neutrophil cells isolated from mouse bone marrow were activated with 1 μM fMLP and migrated on silicon surfaces and observed by field emission scanning electron microscopy. Scale bar, 2 μm. Boxed regions in the right down is an enlarged migrasome. Scale bar, 200 nm. **f** Intravital imaging of neutrophils in mouse liver. Neutrophils were labeled with PE-anti-Ly6G6C (green), and blood vessels were labeled with AF647-WGA (purple). The white arrow indicates the migrasome. Scale bar, 2 μm. **g** Intravital imaging of mouse circulating monocytes and monocyte-derived extracellular particles. LPS (12 mg/kg) was injected into mice by intraperitoneal injection. Monocytes were labeled with CCR2-PE antibody (yellow), and blood vessels were labeled with AF647-WGA (cyan). Scale bar, 5 μm. **h** Confocal image of migrasomes in zebrafish. A single blastomere of an embryo at the eight-cell stage was injected with tspan4a-GFP mRNA. Spinning disk microscopy was used to acquire 3D images of gastrula cells (6 hpf). **i** Transmission electron microscopy image of CAM of chicken embryos from day 9. Individual migrasomes are pointed by black arrows. Scale bar, 1 μm.

Migrasomes have been observed in a wide variety of cell types (Fig. 1). In cultured cells, they are found in diverse primary cell types, including fibroblasts, endothelial cells, epithelial cells, mesenchymal stem cells (MSCs), neutrophils, and various immortalized cell lines, such as L929, NRK, and multiple cancer cell lines.^1,4,12,24–52^ Some of these cell lines, such as L929 and NRK, are extensively used to study the mechanisms underlying migrasome biogenesis.^1,4,24,26,39,40^ In contrast, certain cells are unable to form migrasomes. These cells typically exhibit minimal migratory behavior under in vitro culture conditions and tend to form clusters. It remains unclear whether this reflects an intrinsic lack of migration, and consequently an inability to form migrasomes, or if it is merely due to culture conditions failing to provide the necessary cues to induce migration and migrasome formation. An important factor for migrasome formation in in vitro culture is the type of substrate coating. With few exceptions, migrasome formation generally requires coating culture plates with specific extracellular matrix (ECM) components, which must match the adhesion proteins expressed by the cells.^24^ Although in vitro culture offers a convenient model for studying the mechanisms of migrasome biogenesis, caution must be exercised when interpreting results. In particular, in vitro systems may introduce artifacts that could confound studies of the functional roles of migrasomes.

Migrasomes have also been observed in various ex vivo and in vivo settings. These include mesodermal and endodermal cells in zebrafish embryos during gastrulation, monocytes in the chicken chorioallantoic membrane (CAM) during embryonic angiogenesis, monocytes, neutrophils, circulating tumor cells, and bacterial toxin-treated endothelial cells and Kupffer cells within blood vessels.^3,5,11,53–61^ Additionally, migrasomes have been identified in natural killer cells in mouse spleen.^10^ After their formation, in vivo generated migrasomes follow distinct fates. Some remain localized at their site of generation, such as monocyte-derived migrasomes in the CAM.^54^ Others are displaced and transported to different regions of the organism. For example, migrasomes generated by mesodermal and endodermal cells accumulate in a cavity beneath the embryonic shield,^53^ whereas those formed in blood vessels are dislodged by blood flow and enter circulation.^3,5^ As we will discuss later, these diverse distribution mechanisms have significant implications for the functional roles of migrasomes.

## Migrasome biogenesis

The term “migrasome” was originally coined based on the observation that migrasomes are generated from migrating cells.^1^ However, recent studies have shown that migrasome formation can also be induced in a migration-independent manner, revealing a non-canonical pathway for their formation. For example, the bacterial toxin *Clostridioides difficile* TcdB3 has been shown to induce dramatic migrasome formation in a migration-independent manner.^58^ Some, but not all, genes essential for canonical migrasome formation are also required for the non-canonical pathway, suggesting that part of the molecular machinery involved in canonical migrasome formation is also necessary for the non-canonical pathway. A key feature of this non-canonical migrasome formation is the retraction of the cell body, which generates relative movement between the cell edge and the ECM to which it is attached. This mechanism is similar to the rear edge movement during migration, where both processes create relative movement with the ECM, generating mechanical forces that pull out retraction fibers. These findings suggest that migrasomes can form in stationary cells when induced by specific, physiologically relevant cues. They significantly broaden the concept of migrasome formation, extending it from migrating cells to stationary cells.

For the migration-dependent, canonical migrasome pathway, it is clear that cell migration plays a major role in migrasome formation. A recent study demonstrated that both the speed and pattern of migration can significantly affect the number of migrasomes formed.^30^ Given that cells can alter their migration patterns in response to different environmental cues, this likely represents an important mechanism for regulating migrasome formation in vivo.

### Canonical pathway

**Fig. 2:**
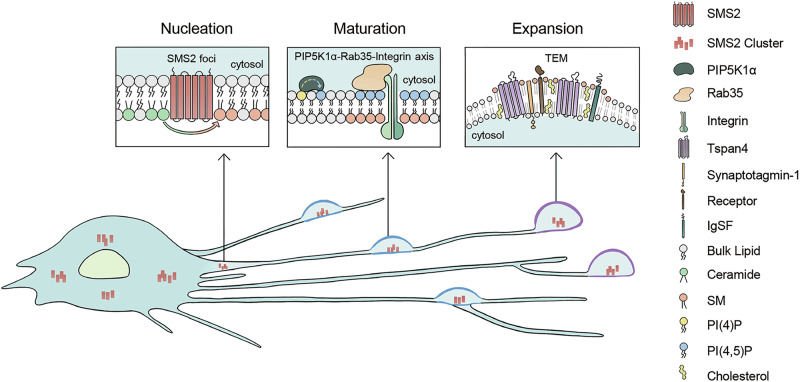
Mechanism of migrasome biogenesis. Migrasome biogenesis occurs in three distinct stages: the nucleation phase, triggered by the assembly of SMS2 foci; the maturation phase, orchestrated by the PIP5K1α-Rab35-integrin signaling axis; and the expansion phase, facilitated by the formation of TEMs.

Studies on migrasome formation in migrating cells reveal that the process is surprisingly complex and can be divided into three major phases: nucleation, maturation, and expansion. Each phase exhibits distinct biochemical, biophysical, and morphological characteristics (Fig. 2).

### Nucleation

Migrasomes are formed on retraction fibers, which are generated at the rear of a migrating cell, leading to the assumption that site determination occurs at the retraction fibers or the back of the cell. However, recent studies have shown that the nucleation of migrasomes is actually determined at the front of the cell.^40^ This study identified sphingomyelin (SM) as being highly enriched in migrasomes and essential for their formation and maintenance of structural integrity. Furthermore, sphingomyelin synthase 2 (SMS2), a transmembrane protein localized to the plasma membrane that converts ceramide into SM, was found to be a key factor in determining migrasome formation sites. SMS2 clusters into puncta on the basal membrane at the leading edge of the migrating cell and remains stationary relative to the substratum, likely through direct binding via its extracellular domain. As the cell moves forward, these SMS2 foci, which are adhered to the substratum and cannot move, appear to “move” to the rear of the cell, eventually reaching the retraction fiber. There, SMS2 catalyzes the conversion of ceramide into SM, promoting migrasome formation by aiding the formation of tetraspanin-enriched macrodomain (TEMA), as will be discussed further.

### Maturation

The conversion of ceramide to SM at the nucleation site sets the stage for the next step in migrasome formation. Concurrently, a series of signaling events occur at the nucleation site, transforming it into a migrasome formation site during the maturation stage. A key player in this maturation process is phosphatidylinositol(4,5)bisphosphate (PI(4,5)P2), which is involved in various signaling pathways by recruiting proteins with PI(4,5)P2-binding domains and triggering the downstream recruitment of binding proteins within the signaling hub.^39^ During migrasome maturation, phosphatidylinositol-4-phosphate 5-kinase type I α (PIP5K1α), a kinase that converts phosphatidylinositol-4-phosphate (PI4P) into PI(4,5)P2, is recruited to the nucleation sites, leading to de novo synthesis and local accumulation of PI(4,5)P2. PI(4,5)P2 then recruits the small GTPase Rab35, which binds to the GFFKR motif of integrin α5. This interaction results in the recruitment of both Rab35 and integrin α5, ensuring that integrins and the ECM serve as a tight anchor, providing a mechanically stable platform for migrasome expansion. Given the prevalence of PI(4,5)P2-binding proteins, it is possible that PI(4,5)P2 may also regulate other aspects of migrasome formation through interactions with its binding proteins in addition to Rab35.

### Expansion

During the formation of retraction fibers, the retraction fiber network is dynamically rearranged, and the junctions are usually stabilized at the sites with stronger adhesion to ECM.^24^ Thus, the strong adhesion at migrasome formation sites provided by PI(4,5)P2-Rab35-integrin axis would assist the junction formation and stabilization.^39^ This provides a stage for the initial swelling of migrasome formation sites, driven by membrane tension fluctuation. When a cell migrates forward, the lateral tension on retraction fibers is highly dynamic. Recent studies suggest that membrane tension fluctuation on retraction fibers is an essential trigger in morphological transition at migrasome formation sites.^62^ Using biomimicking system combined with living cell imaging analysis, researcher founds that during the generation of a retraction fiber, force generated by cell migration would induce dynamic fluctuation of the membrane tension, resulting in the well-understood phenome known as pealing instability observed in elongated structures, such as cylindrical membranes or filaments, where periodic “pearls” or bulges form along the length of the structure. This instability occurs when there is a mismatch between the surface tension and the internal pressure of the structure, causing the structure to break up into spherical or bead-like segments. Pealing instability results in swelling of sections of retraction fiber into small bulge, and some of them eventually row into migrasomes. These swellings often occur at the three-way junctions of retraction fiber. Recent work has shown that when a junction is created on the membrane tether and the membrane tension is increased, the migrasome-like swelling preferentially appears at the junction, as when the membrane tension is the same, junctions are more likely to deform than tubules because of geometric differences.^27^

Tension fluctuation-induced swelling at the junctions or ends of retraction fibers is highly unstable and transient, necessitating stabilization mechanisms to transform these small swellings into migrasomes. Current evidence suggests that multiple mechanisms provide stepwise stabilization, gradually increasing the stability of the swellings and facilitating their transformation into mature migrasomes. A recent study found that Synaptotagmin-1 (Syt1), a well-known calcium sensor, can stabilize these transient swellings, leading to the formation of larger migrasome precursors in a calcium-dependent manner.^31^ These unstable precursors are further stabilized by the recruitment of tetraspanins, marking the expansion stage.

Tetraspanins, a family of transmembrane proteins, organize into tetraspanin-enriched microdomains (TEMs) that play key roles in cell signaling, adhesion, and migration.^63–65^ During the expansion stage, discrete TEMs are recruited to the migrasome precursors and assemble into larger, cholesterol- and SM-rich TEMAs.^26^ While the assembly process is not fully understood, SM likely acts as a “glue” to hold the TEMAs together.^40^ TEMAs exhibit elevated membrane-bending rigidity due to the high enrichment of Tspan4 and cholesterol, which promotes migrasome expansion in two ways. First, when a membrane tether is stretched, the rigid sections resist thinning, leading to swelling. As these rigid sections move laterally and assemble into larger rigid regions, the bulge grows. Second, the increased membrane-bending rigidity further stabilizes migrasomes and establishes a barrier for material exchange, limiting molecule diffusion and preventing migrasome shrinking.^26^

## Mechanisms for cargo loading and release

**Fig. 3:**
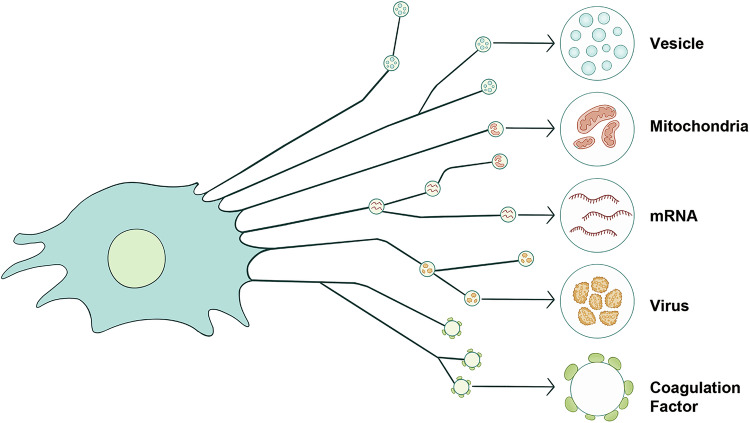
Cargoes of migrasomes. The cargoes carried by migrasomes can be classified into five categories: secretory vesicles, damaged mitochondria, mRNA, viruses, and coagulation factors.

Migrasomes contain a wide variety of cargo, including subcellular structures, virus particles, proteins, lipids, and nucleic acids (Fig. 3). Emerging evidence indicates that these cargoes are actively loaded into migrasomes via specific mechanisms, and their release is similarly governed by distinct regulatory processes. Depending on the loading mechanism, cargoes localize to distinct regions of the migrasome. For example, proteins such as coagulation factors adhere to the surface of neu-migrasomes, while secretory vesicles, damaged mitochondria, MVBs, virus particles, cytosolic proteins, and mRNAs reside within the migrasome lumen.^1,3,4,25,28,37,38,42,50,53^ Secretory proteins, in turn, are encapsulated within secretory vesicles that are themselves localized inside migrasomes.^4^ This diverse topological organization of migrasome cargo likely reflects the functional requirements of each cargo type and is intricately linked to the mechanisms governing cargo loading and release.

### Cargo-loading mechanisms

Multiple subcellular structures, including secretory vesicles, damaged mitochondria, and MVBs, are transported into migrasomes.^4,25,38^ While the mechanisms for mitochondrial and MVB transport remain unclear, how secretory vesicles traffic into migrasomes has been elucidated. Migrasomes are characterized by ILVs derived from distinct trafficking pathways: Rab8/VAMP2-positive vesicles from constitutive secretion, Rab11/VAMP3-positive vesicles from recycling endosomes, and Rab10/CAV1-positive vesicles from other pathways.^4,12^ In stationary cells, these vesicles are non-specifically transported to the plasma membrane for exocytosis. During migration, however, myosin 5a redirects them to the base of retraction fibers, and actin bundles in retraction fibers then serve as transport routes, with myosin 5a facilitating vesicle transportation into migrasomes.^4^ This mechanism likely plays an important role in mediating the physiological functions of migrasomes, as secretory proteins with signal peptides — including many ligands critical for physiological processes — are selectively and actively transported into migrasomes via this pathway.^5^

Less is known about how other subcellular structures are transported into migrasomes. For damaged mitochondria, although the precise mechanisms remain to be determined, current evidence suggests that motor proteins likely play important roles.^25^ For example, microtubule-based motor protein KIF3A has been shown to be required for the peripheral localization of damaged mitochondria, while myosin 19, a mitochondrial-binding myosin, is required for efficient transport into migrasomes.^25^ MVB and virus particle transport may also involve motor proteins, as these structures are observed in migrasomes connected to retraction fibers containing actin and microtubules.^38^

Cargoes can also be loaded onto the outer surface of migrasomes through absorption from the environment. Recent studies demonstrate that neu-migrasomes are enriched with coagulation factors, which are not produced by neutrophils but secreted by the liver into the bloodstream. Once neu-migrasomes are released into circulation, these factors are specifically adsorbed onto their surface. This absorption is highly selective, as platelets — despite sharing a similar size and circulatory environment — do not adsorb these factors. Protease K treatment removes adsorbed coagulation factors from neu-migrasomes, but re-incubation with serum restores their adsorption, suggesting a lipid-mediated mechanism. Liposomes mimicking neu-migrasome lipid composition, but not those resembling platelet or neutrophil cell membrane lipids, successfully adsorb coagulation factors, indicating that specificity arises from the unique lipid profile of neu-migrasomes.^3^

The mechanism by which cytosolic components, such as mRNAs and cytosolic proteins, are loaded into migrasomes remains entirely unknown. However, the striking specificity of their enrichment strongly implies the existence of a tightly regulated process. For instance, migrasomes selectively accumulate only a small subset (~200) of mRNAs out of the thousands present in the cell.^28^ Similarly, cytosolic proteins enriched in migrasomes exhibit distinct molecular signatures compared to the broader cytoplasmic pool.^5,7,53^ The observed selectivity — evident in both nucleic acids and proteins — implies the existence of active, discriminative cargo-loading systems. These mechanisms, though not yet characterized, likely involve precise molecular recognition or sorting pathways to achieve such specificity.

### Cargo release mechanisms

Upon entry into migrasomes, cargoes must be released to execute their functions. Current evidence indicates that secretory proteins are discharged through membrane fusion between secretory vesicles and migrasomes, a process regulated by SNARE complexes: R-SNAREs (VAMP2/3) on secretory vesicles interact with Q-SNAREs (SNAP23) on the migrasome membrane.^4^ This fusion is tightly regulated — for example, calcium signaling within migrasomes appears critical, as depletion of extracellular calcium blocks secretory vesicle exocytosis, trapping cargoes inside migrasomes. Such fusion mechanisms may not be limited to secretory vesicles. For instance, MVBs within migrasomes could theoretically fuse with the migrasome membrane, potentially releasing exosomes or other luminal components.^38^ However, this hypothesis remains speculative and necessitates experimental validation.

Cytosolic cargoes, such as proteins and mRNAs, likely employ distinct release mechanisms compared to vesicle-bound secretory cargoes. Notably, migrasomes have been observed to develop membrane permeability during late stages of biogenesis.^28,53^ This leakiness raises the possibility of passive cytosolic cargo escape, potentially serving as an alternative pathway for non-vesicular components. For example, cytosolic proteins or mRNAs trapped within migrasomes could diffuse into the extracellular space as membrane integrity declines, bypassing fusion-dependent mechanisms. However, whether this leakage represents a regulated process or a degenerative byproduct remains unclear. Intriguingly, such timed permeability might align with migrasome functional roles, enabling staggered cargo release — first via controlled vesicle fusion, followed by bulk leakage of residual cytosolic materials. Nevertheless, direct evidence linking leakiness to functional cargo release is lacking, and further studies are needed to validate this hypothesis.

## Physiological functions of migrasomes

**Fig. 4:**
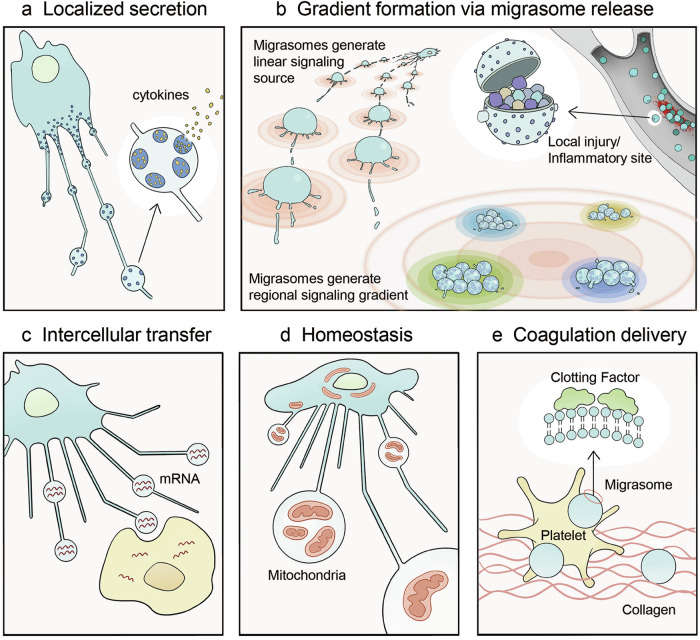
Biological functions of migrasomes. Migrasomes participate in diverse physiological processes through multiple mechanisms: **a** localized secretion of signaling molecules; **b** targeted delivery of signaling cues via detached migrasomes, contributing to the formation of spatially defined signaling centers and gradients during embryonic development, angiogenesis, and immune responses; **c** intercellular transfer of proteins and RNAs between neighboring cells, fostering cell–cell communication; **d** maintenance of cellular homeostasis under stress conditions by expelling damaged organelles; **e** absorption and targeted delivery of coagulation factors to promote clotting.

In multicellular organisms, cells do not operate in isolation but rely on tightly orchestrated signaling networks to coordinate behaviors — from migration and nutrient uptake to growth, division, and specialized functions. These signals, including ligands such as growth factors, cytokines, chemokines, and bioactive molecules (e.g., coagulation factors), must be delivered with spatiotemporal precision to ensure seamless intercellular coordination. Migrasomes have emerged as critical mediators of this system-wide signaling, enabling cells to communicate in a spatially defined, temporally controlled, and activity-specific manner.^3,5,53,54^ By serving as dynamic signaling hubs, migrasomes facilitate the targeted delivery of molecular cues, ensuring that ligands reach their intended destinations at the right time and in the correct combinatorial context (Fig. 4).

Moreover, migrasomes bridge cell migration with broader biological processes. Migration itself is rarely an isolated event; it is intricately linked to developmental morphogenesis, immune surveillance, tissue repair, and other processes where concurrent cellular activities — proliferation, differentiation, secretion, homeostasis, and even cell death — must be harmonized. Migrasomes act as a linchpin in this integration, coupling migratory behavior with the regulation of these diverse functions.^3,5,53,54^

It is important to note that the function of migrasomes is intrinsically linked to their structure and composition. Not only does the cargo influence the function of migrasomes, but other structural elements likely contribute as well. For example, migrasomes are formed by the assembly of TEMs, which are known to be highly enriched with adhesion molecules such as integrins, immune-modulating molecules like MHC, co-stimulatory molecules, and receptors for ligands.^63–65^ Understanding the relationship between structure and function may provide valuable insights when investigating the physiological roles of migrasomes.

### The cellular functions of migrasomes

To decipher the physiological roles of migrasomes, we must first understand their functions at the cellular level. These functions are intrinsically tied to their cargo loading and release mechanisms (discussed earlier). Current evidence categorizes migrasome functions into four key roles. 1) Ligand source for signaling: migrasomes deliver specific ligands to spatiotemporally defined locations, regulating processes such as embryonic development.^5,53,54^ 2) Vehicle for lateral transfer: migrasomes produced by one cell are taken up by others, enabling material and information exchange between cells.^1,28^ 3) Cellular homeostasis mechanism: damaged organelles, toxic substances, or unwanted materials are disposed of via migrasomes.^25^ 4) Molecular absorption platform: migrasomes adsorb molecules from their surroundings, loading their surface with components secreted by other cells (e.g., liver-derived coagulation factors on neu-migrasomes).^3^ These cellular functions form the foundation for the physiological roles of migrasomes. Through these functional modules, migrasomes from individual cells can exert influence across the entire organism.

### Context-dependent physiological functions of migrasomes

The physiological relevance of migrasomes must also be contextualized within broader biological systems. Like many cellular systems, their roles are context-dependent. A parallel example is autophagy — a lysosome-based degradation system — which exhibits diverse physiological functions depending on cell type, substrate, and biological process.^66^ Similarly, migrasomes primarily function in the release and targeted delivery of cellular contents; their physiological impact hinges on what is released and delivered, when it occurs, and the specific cellular or tissue context. To date, migrasomes have been primarily studied in embryonic development and immune regulation — contexts rich in cell migration and communication.^3,5,53,54^ Given the ubiquity of these processes across biology, migrasomes likely play roles in other settings, such as tissue repair, cancer metastasis, or metabolic regulation, though these remain underexplored.

### Migrasome in systemic signaling

Signaling molecules, such as morphogens, cytokines, chemokines, and growth factors, play critical roles in a wide range of biological processes. The current framework for understanding their spatial and temporal regulation relies on the concept of signal gradients.^67–70^ Gradients are established through the localized production of signaling molecules, which diffuse outward and decrease in concentration with distance. Their profiles are refined by processes such as degradation, receptor uptake, and binding to the ECM, while physical barriers like membranes or tissues further shape their distribution. This model is often considered as a “passive” mechanism, with gradients emerging in a manner that does not necessarily involve active temporal-spatial control.

Recent research on migrasomes reveal their important role in actively delivering the right signaling molecules to the right place at the right time. Three scenarios for this targeted delivery have been described. In zebrafish, during early embryonic development, migrasomes are produced by mesodermal and endodermal cells and are enriched with Cxcl12, a chemokine essential for organ morphogenesis. These migrasomes are released from the cells and accumulate in a cavity beneath the embryonic shield. There, they act as a localized source of Cxcl12, attracting dorsal foreman cells to the embryonic shield. The dorsal foreman cells then organize into Kupffer’s vesicle, a temporary organ in zebrafish crucial for organ development. Without migrasomes, dorsal foreman cells fail to migrate correctly to the embryonic shield, disrupting Kupffer’s vesicle formation and hindering organ development.^53^ In this context, migrasomes provide a spatially defined, localized signaling cues for proper development.

In the second scenario, within the CAM, monocytes deposit migrasomes along their migratory paths during angiogenesis. These migrasomes are highly enriched with angiogenic factors, such as vascular endothelial growth factor A (VEGF-A), forming a linear trail of VEGF-A-enriched migrasomes. Tip cells, which are specialized endothelial cells at the forefront of growing blood vessel sprouts, detect and respond to VEGF-A gradients, guiding the sprout to follow the monocyte’s migratory trajectory. This mechanism, referred to as the “Vanguard model” of angiogenesis, highlights the role of monocytes as pattern planners for embryonic blood vessel formation, with migrasomes acting as critical mediators for both stimulating angiogenesis and defining the spatial pattern of the developing vasculature.^54^

The third scenario involves the formation of a localized gradient within high-velocity flow systems, such as the bloodstream. Following lipopolysaccharide (LPS) treatment, monocytes produce migrasomes enriched with pro-inflammatory cytokines, including TNF-α and IL-6, which are deposited on the endothelial surface of blood vessels. These migrasomes are subsequently washed into circulation. Their surfaces are enriched with adhesion molecules, enabling them to interact with activated endothelial cells during local inflammation. Endothelial activation, marked by the upregulation of selectins and integrins, facilitates the rapid accumulation of circulating migrasomes at inflamed sites. Although the precise mechanism is not yet fully understood, these migrasomes likely “sense” inflammation through enriched adhesion molecules and deliver cytokine cargoes to inflamed regions, creating a localized concentration gradient within the high-velocity flow.^5^

In addition to secretory proteins, migrasomes can also redistribute bioactive molecules. A key example is the recent discovery that neu-migrasomes are essential part of coagulation system. Coagulation factors, which are primarily secreted from the liver into the bloodstream at low concentrations to prevent unregulated clotting, can accumulate on the surface of neu-migrasomes. Compared to platelets, the concentration of coagulation factors is much higher on migrasomes. When an injury exposes collagen, neu-migrasomes accumulate at the site through interactions with collagen. At the same time, platelets gather at the injury site, where coagulation factors on the migrasomes activate platelets to trigger clotting. This mechanism allows low levels of coagulation factors in the blood to be pre-packaged on migrasomes, forming a binary system with platelets. These components, normally compartmentalized, accumulate at the injury site to ensure proper clotting while preventing abnormal coagulation under normal conditions.^3^

As a signaling distribution system, migrasomes offer several advantages. First, a single migrasome can carry a combination of signaling molecules, enabling the delivery of complex combinatorial signals that convey more information than individual molecules.^3,5,53^ Second, migrasomes can be distributed throughout the organism via different mechanisms, including long-range transport through the bloodstream or localized deposition along migratory pathways.^3,5,54^ This allows for both system-level delivery and the formation of intricate spatial signaling patterns. Finally, migrasomes can bind to regions with elevated expression of adhesion molecules, enabling them to “sense” abnormalities such as local inflammation or injury, where adhesion molecule levels are upregulated.^3,5^ Collectively, these characteristics position migrasomes as a flexible, versatile, and efficient mechanism for regulating signaling events at a systemic level.

## Migrasomes in cellular homeostasis

In addition to systemic signaling, migrasomes have been reported to play a role in maintaining cellular homeostasis. During mild mitochondrial stress, damaged mitochondria can be evicted from the cell via migrasomes. This process, referred to as mitocytosis, ensures the timely clearance of damaged mitochondria, preventing the accumulation of toxic mitochondria in migrating cells. In long-migrating cells, such as neutrophils circulating in blood vessels, mitocytosis appears crucial for maintaining cellular vitality, possibly by preventing the buildup of damaged mitochondria.^25^ At this point, the prevalence of this mechanism in the clearance of damaged or toxic cellular structures remains unclear, and further research is needed to explore this intriguing process.

### Migrasome in diseases

As our understanding of the physiological role of migrasomes continues to grow, their potential involvement in various diseases is becoming increasingly evident. However, at this stage, our knowledge of migrasomes in disease contexts remains preliminary, often indirect, and inconclusive. The existing reports can be categorized into three key areas: first, implication of genes essential for migrasome formation in various diseases, which may suggest a link between migrasomes and these diseases, but could also reflect the migrasome-independent roles of these genes; second, the association between migrasome formation and disease, with the implication that migrasomes could serve as potential diagnostic or prognostic markers; and third, more established cases where causal evidence supports the direct involvement of migrasomes in disease processes. Together, these direct and indirect pieces of evidence suggest that migrasomes may be involved in a wide range of diseases, particularly cancer, cardiovascular conditions, immune regulation, and infectious diseases.

Numerous studies have focused on the association between genes essential for migrasome formation and various diseases. In a recent study, the expression, prognosis, genetic variation, and drug sensitivity profiles of migrasome-related genes (MRGs) were analyzed across pan-cancer datasets. A migrasome score was constructed using gene set enrichment analysis, revealing its significant role in tumor development, immune escape, and prognosis, with high migrasome expression linked to poor prognosis and immune-related markers. The migrasome score correlated significantly with tumor immunity and stroma scores, macrophage abundance, and immune checkpoint genes.^71^ Additionally, in cardiovascular research, a migrasome-related signature was developed using machine learning to predict acute myocardial infarction (AMI), identifying ITGB1 as a key gene in macrophage polarization and suggesting ginsenoside Rh1 as a potential therapeutic agent.^72^ Individual migrasome essential genes, such as Tspan4, have also been linked to various diseases. While these findings provide valuable insights into the potential link between migrasomes and diseases, further direct evidence is needed.^73–75^

Migrasome formation has been observed in various physiological and pathological processes, suggesting their potential role as mediators of disease progression and as promising biomarkers and therapeutic targets.^76–78^ Recent studies have shown that neu-migrasomes are essential for the coagulation system, while monocyte-derived migrasomes are enriched with pro-inflammatory cytokines. Significant increases in both neutrophil- and monocyte-derived migrasomes in LPS-treated mice suggest their potential involvement in inflammation-related disease progression, including abnormal clotting and excessive cytokine release.^3,5^ In kidney disease, podocytes release migrasomes upon injury, and elevated urinary migrasome levels serve as a sensitive, non-invasive biomarker for early podocyte damage, providing a potential alternative marker for detecting kidney dysfunction.^33^ Additionally, a method using wheat germ agglutinin (WGA)-coated magnetic beads and flow cytometry has been developed to capture and quantify migrasomes, further supporting their diagnostic potential in kidney disease.^79^ While these studies provide a more direct link between migrasomes and diseases, it is important to note that the causal relationship between migrasome formation and these diseases still needs to be established.

Recent studies have demonstrated that migrasomes can directly elicit pathological effects, with migrasomes derived from specific cell types being induced in disease settings and contributing to disease progression.^29,35,36,38,41,46,49,59–61,80^ For example, during bone metastasis, tumor cells trigger the differentiation of RANKL-activated osteoclast precursors into osteoclasts in a migrasome-dependent manner. This process, known as tumor-induced osteoclast coupling, relies on the migrasomal transfer of tumor cell cytoplasmic material. This insight has led to the development of a nanoliposome-based therapy that inhibits migrasome formation, decouples the tumor–osteoclast interaction, and induces cell death, thereby offering a potential strategy for early prevention of bone metastasis.^44^ In a cerebral amyloid angiopathy model, Aβ40 induces migrasome formation in macrophages, and these CD5L-enriched migrasomes bind to blood vessels and contribute to blood–brain barrier damage, with complement activation playing a key role in the process.^57^ Furthermore, genetic evidence suggests that impairing migrasome formation can directly influence disease outcomes. For instance, the bacterial toxin *Clostridioides difficile* TcdB3 induces migrasome production in liver endothelial cells and Kupffer cells in vivo.^58^ Migracytosis-defective *Tspan9*^*−*/*−*^ mice, which exhibit reduced migrasome formation, show less acute inflammation and lower lethality in toxin challenge assays.^5^ These findings highlight non-canonical migracytosis as a novel mechanism by which mammals sense and exacerbate early immune responses during microbial infections.

A series of recent studies have focused on the role of migrasomes in viral infections.^47^ Specific viral proteins, such as Chikungunya virus nsP1, have been shown to induce migrasome formation in infected cells.^37^ Moreover, Vaccinia virus infection has been demonstrated to trigger migrasome formation, with some migrasomes containing the viral particles.^43^ This observation led the authors to speculate that migrasomes may represent a novel mechanism for poxvirus spread. In a subsequent study, the same group reported that tecovirimat/ST-246, the current treatment for mpox virus, does not block infection-induced migrasome formation or the loading of virions into migrasomes, suggesting that this mechanism may enable mpox virus to evade ST-246 treatment.^50^ In a later study, the group used Dasabuvir, an FDA-approved hepatitis C virus inhibitor, which also inhibits ROCK1, a known migrasome regulator. They found that Dasabuvir effectively inhibits poxvirus infection-induced migrasome formation and disrupts the formation of extracellular enveloped virus, preventing the release of vaccinia virus from infected cells.^52^ This provides further evidence for migrasome-mediated virus spread. Direct evidence of migrasome-mediated virus spread was reported when cells infected with herpes simplex virus type 2 released migrasomes containing viral particles, which could be transmitted to uninfected cells and cause productive infection.^42^ While these studies were conducted in vitro, they suggest that migrasomes may play a role in virus spreading, a hypothesis that should be tested in in vivo infection models in the future.

## Methods for studying migrasomes

A broad range of methodologies has been developed for studying migrasomes. These include advanced imaging techniques that allow visualization of migrasomes in vivo and in vitro, as well as protocols for isolating and characterizing migrasomes from various biological samples.^10,11,55,56,81^ Quantitative methods, employing biochemical assays, imaging, and flow cytometry, have been established to accurately measure migrasome abundance and dynamics.^3,5,7^ Furthermore, various animal models have been developed to facilitate the investigation of their cellular and physiological roles.^3,4,25,44,53,54^ Together, these methodological advancements provide a robust toolkit for exploring the formation, regulation, and function of migrasomes in both health and disease.

Live imaging is an essential tool for studying migrasome biogenesis because it enables visualization and quantification of key events, as well as determining when and where migrasomes are generated in vivo — providing important clues about their physiological roles. Migrasomes are typically labeled using genetically encoded fluorescent proteins or chemical probes. For example, fluorescent tags such as GFP, RFP, and mCherry fused to proteins enriched in migrasomes — like the PI(4,5)P2-binding domain PH-PLCδ, tetraspanin family proteins (e.g., TSPAN4), and, to a lesser extent, integrin α — are widely used for live imaging.^1,24,39^ It is important to note that overexpression of these fluorescently tagged markers can alter migrasome biogenesis; for instance, TSPAN4-GFP overexpression dramatically enhances migrasome formation, whereas overexpression of PH-PLCδ does not, making it a potentially better marker in some contexts.^26,39^ Additionally, fluorescently tagged markers have limitations: they can be time-consuming to use and difficult to transfect into many cell types, especially primary cells. As an alternative, live cell staining with dyes — such as GFP-tagged non-toxic lysenin (NT-Lys) for clustered SM,^40^ fluorescently tagged WGA for glycoproteins and glycolipids,^82^ and the amphiphilic rhodamine probe RMG3 for lipid bilayers^83 ^— provides a rapid, simple, and non-interfering method for migrasome study. Moreover, fluorescently conjugated antibodies that target proteins enriched on the surface of migrasomes offer an effective method for observing these structures in specific cell types. For instance, anti-Ly6G can be used to label migrasomes in neutrophils, and anti-CCR2 is effective for monocytes, both in vivo and in vitro.^3,5^

Migrasomes can be isolated from both in vitro cultured cells and various in vivo samples, including biofluids like blood and urine, as well as relatively simple, loosely organized samples such as zebrafish embryos during gastrulation and CMA from chicken embryos.^1,3,7,33,53^ However, effective protocols for isolating migrasomes from densely organized tissues, such as solid tumors, are still lacking because tissue dissociation often results in cell rupture and the release of intracellular structures similar in size to migrasomes, making high-purity isolation challenging. For migrasome isolation from cultured cells, differential centrifugation and density gradient centrifugation are commonly used and are effective in separating migrasomes from small EVs (e.g., exosomes) and cellular debris.^7,81^ In contrast, for complex in vivo samples like blood, antibody-based methods are preferred. Typically, cells are first removed by low-speed centrifugation, and large EVs, including migrasomes, are pelleted using higher-speed centrifugation that does not sediment smaller EVs. Finally, positive or negative selection with specific antibodies (e.g., targeting neutrophils) can be used to isolate migrasomes from the desired cell type.^3,5,81^ Recent studies indicate that the majority of large EVs isolated from neutrophils from healthy mouse blood by these methods are migrasomes.^3^ Once isolated, migrasomes can be identified using a panel of markers, while simultaneously checking for markers of other EV types and organelles to monitor potential contamination.^3,7,81^

Quantification of migrasomes can be accomplished using imaging, flow cytometry, and biochemical approaches, each with its own advantages and limitations. High-resolution imaging allows precise quantification of migrasomes within individual cells and provides detailed morphological information; however, it is limited by the number of cells that can be analyzed and by the restricted observation window available for in vivo studies (e.g., only certain regions, such as blood vessels on the liver surface, may be accessible).^3,5^ Flow cytometry enables rapid analysis of large sample sizes, but the small size of migrasomes and the presence of similarly sized particles in biological samples can lead to artifacts. Imaging flow cytometry, which combines the strengths of both techniques, offers improved morphological insights but is generally less accessible than standard flow cytometry.^3,5^ Biochemical analyses can assess multiple markers simultaneously and help rule out contamination from other EVs, although not all EV types have well-defined markers.^3,5,7,54^ Consequently, employing a combination of these methods is recommended to achieve the most reliable quantification of migrasomes.

Various ex vivo and in vivo models have been employed to study migrasomes, providing key insights into their physiological roles. One of the main considerations when developing these models, especially in the early stages of migrasome research, is whether they allow for direct observation using microscopy. Thus, model systems that are amenable to microscopy studies have been prioritized, including zebrafish embryos, chick CAM, and, more recently, mice, as in vivo observation of mouse migrasomes has become possible.^3,5,53,54^ Establishing genetic models in which key migrasome genes are knocked out or knocked down enables researchers to dissect the physiological roles of migrasomes more precisely. In comparison to other cellular processes, migrasomes offer more direct evidence for establishing causal relationships due to their unique properties. Research on the physiological functions of other basic cellular processes often faces challenges in unraveling the complex relationships between genes, cellular functions, and physiological outcomes. For instance, gene knockout may impair cellular processes and physiological functions simultaneously, complicating the determination of whether the observed effects are causal or merely parallel. In contrast, migrasomes can often be reintroduced into gene knockout animals to directly assess their function. If the phenotype is rescued upon reintroduction, it provides clear evidence that the physiological effect is independent of the other gene functions, thereby confirming a direct causal relationship between the migrasome and the observed physiological function.^3,53,54^

These methodological developments encourage researchers to adopt a more comprehensive approach to study migrasomes, which could include steps such as (1) observing and quantifying migrasomes microscopically whenever possible to capture their dynamics in vivo or in relevant model systems; (2) validating these observations with independent methods, such as biochemical characterization, to confirm the presence and behavior of migrasomes; (3) generating genetic models with impaired migrasome formation, followed by rescue experiments to assess the resulting physiological phenotypes and understand the functional significance of migrasomes; (4) analyzing the cargo associated with migrasomes to gain mechanistic insight into how migrasomes regulate the observed phenotypes; and (5) testing the resulting insights from cargo and phenotype analyses through a combination of cell biology and biochemical studies. By integrating these strategies, researchers can increase the robustness of their findings, providing a more reliable framework for understanding the physiological role of migrasomes.

## Challenge and future direction

A decade has passed since the discovery of migrasomes, and research on their biogenesis, physiological functions, and relevance to disease has transformed what was initially a curious observation into an emerging field of study. However, despite this progress, the nascent field still faces significant challenges as it continues to develop.

Current methods for detecting, isolating, and analyzing migrasomes remain inadequate. Microscopic observation of migrasomes in vivo is limited to a set of narrow windows, with restricted depth and field of view, leaving much of the tissue inaccessible for analysis. Advancing microscopy technology to enable deeper, broader, and more flexible imaging is crucial. Similarly, current labeling methods, particularly for in vivo studies, rely heavily on antibodies that recognize proteins enriched on the migrasome surface. However, antibody-based labeling carries the risk of artifacts due to its interactions with various cells and proteins in vivo. A more reliable approach is to develop animal models in which fluorescent tags are knocked in to label migrasome marker proteins, ensuring precise labeling without interfering with migrasome formation or function.

Another major challenge is the isolation of migrasomes. While antibody-based methods can achieve relatively high purity when isolating migrasomes from specific cell types, they rely on knowledge that, under defined conditions, a particular cell type predominantly produces migrasomes rather than other EVs. This approach is only applicable in limited cases, such as neu-migrasomes in the blood of healthy mice, where the large EV fraction has been shown to consist predominantly of migrasomes.^3^ The lack of migrasome-specific surface markers distinct from other large EVs prevents the development of highly specific isolation techniques. Identifying migrasome-specific markers and designing isolation and analytical methods based on them is therefore an urgent priority. Additionally, current protocols for isolating migrasomes from solid tissues — such as organs and tumors — are inadequate, hindering studies on their physiological functions in these environments. Addressing these methodological gaps is critical for advancing migrasome research and its applications in physiology and disease.

Beyond methodological challenges, fundamental questions about migrasomes remain unanswered. Some of these gaps arise due to technical limitations, while others persist simply because they have been overlooked. Answering these questions could significantly enhance our understanding of migrasomes. Key unknowns include the cellular origins, abundance, distribution, dynamics, fate, and half-life of migrasomes in vivo. Mapping these parameters systematically will likely require a collective effort from multiple research groups over time. However, some aspects, such as the behavior of migrasomes in the circulatory system, may be mapped relatively quickly using targeted studies. Establishing such a “migrasome atlas” would provide a foundation for understanding their physiological roles and disease relevance.

Given that shedding migrasomes leads to significant cell mass loss, their formation is likely to be tightly controlled. Current research supports this idea, yet most studies on migrasome biogenesis have focused on the underlying molecular machinery rather than the regulatory mechanisms governing their formation. Understanding migrasome regulation within the conceptual framework of signal transduction is necessary. This includes identifying the extrinsic and intrinsic factors that trigger migrasome formation, how these signals are recognized and processed by cells, and how they ultimately activate the core migrasome biogenesis machinery. Deciphering these regulatory mechanisms would not only enable the development of more precise genetic models for studying physiological functions but also open possibilities for targeted manipulation of migrasome biogenesis for therapeutic applications.

Current evidence supports the idea that migrasomes play a significant role in a wide range of diseases. The evolving understanding of their physiological functions provides a valuable framework for investigating their disease relevance. Recent studies on migrasomes in the immune system suggest that they may have far-reaching effects, given the central role of immune function in the progression of most, if not all, pathological conditions. Diseases directly caused by immune dysfunction, such as autoimmune disorders and immunodeficiencies, as well as those where immune regulation is a key factor, including cancer progression and infectious diseases, present promising avenues for further study.

As the field progresses, the therapeutic potential of migrasomes may become increasingly apparent. In theory, migrasomes could be “read” to extract disease-specific information, making them valuable tools for diagnosis and prognosis. Conversely, disease-associated abnormalities in migrasome generation could be “rectified” to restore normal function and treat migrasome-related disorders. Additionally, since migrasomes primarily function as an intercellular communication system at the organismal level, they could be repurposed as vehicles for delivering therapeutic payloads. Breakthroughs in harnessing the therapeutic potential of migrasomes would mark a transformative milestone in the field, as the ultimate impact of a discovery is measured by its real-world applications.
